# Examining Rural and Urban Sentiment Difference in COVID-19–Related Topics on Twitter: Word Embedding–Based Retrospective Study

**DOI:** 10.2196/42985

**Published:** 2023-02-15

**Authors:** Yongtai Liu, Zhijun Yin, Congning Ni, Chao Yan, Zhiyu Wan, Bradley Malin

**Affiliations:** 1 Department of Computer Science Vanderbilt University Nashville, TN United States; 2 Department of Biomedical Informatics Vanderbilt University Medical Center Nashville, TN United States; 3 Department of Biostatistics Vanderbilt University Medical Center Nashville, TN United States

**Keywords:** COVID-19, social media, word embedding, topic analysis, sentiment analysis, Twitter, data, vaccination, prevention, urban, rural, epidemic, management, model, training, machine learning

## Abstract

**Background:**

By the end of 2022, more than 100 million people were infected with COVID-19 in the United States, and the cumulative death rate in rural areas (383.5/100,000) was much higher than in urban areas (280.1/100,000). As the pandemic spread, people used social media platforms to express their opinions and concerns about COVID-19–related topics.

**Objective:**

This study aimed to (1) identify the primary COVID-19–related topics in the contiguous United States communicated over Twitter and (2) compare the sentiments urban and rural users expressed about these topics.

**Methods:**

We collected tweets containing geolocation data from May 2020 to January 2022 in the contiguous United States. We relied on the tweets’ geolocations to determine if their authors were in an urban or rural setting. We trained multiple *word2vec* models with several corpora of tweets based on geospatial and timing information. Using a *word2vec* model built on all tweets, we identified hashtags relevant to COVID-19 and performed hashtag clustering to obtain related topics. We then ran an inference analysis for urban and rural sentiments with respect to the topics based on the similarity between topic hashtags and opinion adjectives in the corresponding urban and rural *word2vec* models. Finally, we analyzed the temporal trend in sentiments using monthly *word2vec* models.

**Results:**

We created a corpus of 407 million tweets, 350 million (86%) of which were posted by users in urban areas, while 18 million (4.4%) were posted by users in rural areas. There were 2666 hashtags related to COVID-19, which clustered into 20 topics. Rural users expressed stronger negative sentiments than urban users about COVID-19 prevention strategies and vaccination (*P*<.001). Moreover, there was a clear political divide in the perception of politicians by urban and rural users; these users communicated stronger negative sentiments about Republican and Democratic politicians, respectively (*P*<.001). Regarding misinformation and conspiracy theories, urban users exhibited stronger negative sentiments about the “covidiots” and “China virus” topics, while rural users exhibited stronger negative sentiments about the “Dr. Fauci” and “plandemic” topics. Finally, we observed that urban users’ sentiments about the economy appeared to transition from negative to positive in late 2021, which was in line with the US economic recovery.

**Conclusions:**

This study demonstrates there is a statistically significant difference in the sentiments of urban and rural Twitter users regarding a wide range of COVID-19–related topics. This suggests that social media can be relied upon to monitor public sentiment during pandemics in disparate types of regions. This may assist in the geographically targeted deployment of epidemic prevention and management efforts.

## Introduction

The COVID-19 pandemic has persisted for over two years. By the end of 2022, more than 100 million people in the United States were infected with COVID-19, with notable disparities [1]. In particular, the cumulative death rate in rural areas (383.5/100,000) has been significantly higher than in urban areas (280.1/100,000) [1,2], a disparity that highlights the need to improve practices in prevention and control [3]. However, the path to improving the situation in rural environments is not evident, partially due to the fact that urban and rural residents have different attitudes about COVID-19 and policies regarding its management. For example, it has been shown that rural residents are less concerned about the coronavirus [4] and are less willing to engage in COVID-19–related prevention behaviors [5,6]. Moreover, political polarization influences the public’s attitude and reaction to the COVID-19 pandemic [7,8].

To date, there have been several studies into the differences between urban and rural sentiment about COVID-19 [4,6,9,10]. However, these studies have mainly relied upon formal surveys, which are limited in their ability to shed light on the matter because they are time-consuming, and the findings (as well as the policies based on them) can become stale in the face of the rapid evolution of the situation [11]. Social media platforms have enabled people to report on their experiences and express their perspectives on COVID-19 on a wide scale. The data generated through social media have been relied upon to study various aspects of health and wellness [12-15], such that it is natural to hypothesize that this large and diverse collection of user-generated data provides opportunities to investigate the differences between urban and rural sentiments. In this paper, we report public sentiment on COVID-19–related topics using data from Twitter, one of the largest social platforms in the United States, with over 200 million daily active users [16].

While topic extraction and sentiment analysis are typical natural language processing tasks, prior research on inferring sentiments about COVID-19 from social media has been limited in several ways. First, prior studies [17,18] have relied on topic modeling techniques, such as latent Dirichlet allocation [19], to identify relevant topics from the collected social media data. However, such methods rely on document-level word co-occurrences to infer topic distribution [20], which leads to poor topic extraction performance for noisy short-text data [21,22]. Second, most studies applied either predefined rules [7,17,23-25], such as VADER (Valence Aware Dictionary and Sentiment Reasoner) [26], or machine learning models to infer sentiment from tweets. While rule-based approaches fail to leverage the contextual information in a specific corpus, which varies by corpus, machine learning approaches [27-32] are hindered by their need for a nontrivial amount of label annotation and training [31,33].

Even if the labeling process can be expedited, to initiate a study with social media data, it is necessary to collect online posts on the topic of interest. The majority of earlier studies in this area applied keyword filtering to collect COVID-19–related tweets [34-37]. However, keyword filtering is hindered by an incompleteness problem that can lead to biased investigations. For example, in one vaccination opinion study [38], tweets were collected using the keyword list “vaccine, vaccination, vaccinate, vaccinating, vaccinated,” which neglected all tweets that used the word “vax.” At the same time, the societal response to the pandemic is constantly evolving, with new keywords being generated at different stages. It is unlikely that one would be aware of all appropriate keywords at any point in time. For instance, in the COVID-19-TweetIDs data set [39], the word “vaccine” was not added to the keyword list until November 2021—one year after vaccines received US Food and Drug Administration emergency use authorization.

In this paper, we investigate differences in the sentiments of urban and rural residents regarding COVID-19 and related topics. To accomplish this task, we introduce a novel approach for COVID-19 sentiment analysis. This approach begins by collecting tweets without any predefined keywords. To identify topics from the brief amount of text in a tweet, the approach leverages word-embedding models and a clustering approach to extract topics related to COVID-19. Our new sentiment analysis approach combines lexicons and semantic information to quantify public sentiment with respect to a specific population of interest regarding COVID-19 and related topics, such as prevention, vaccination, and politics.

## Methods

### Overview

**Figure 1:**
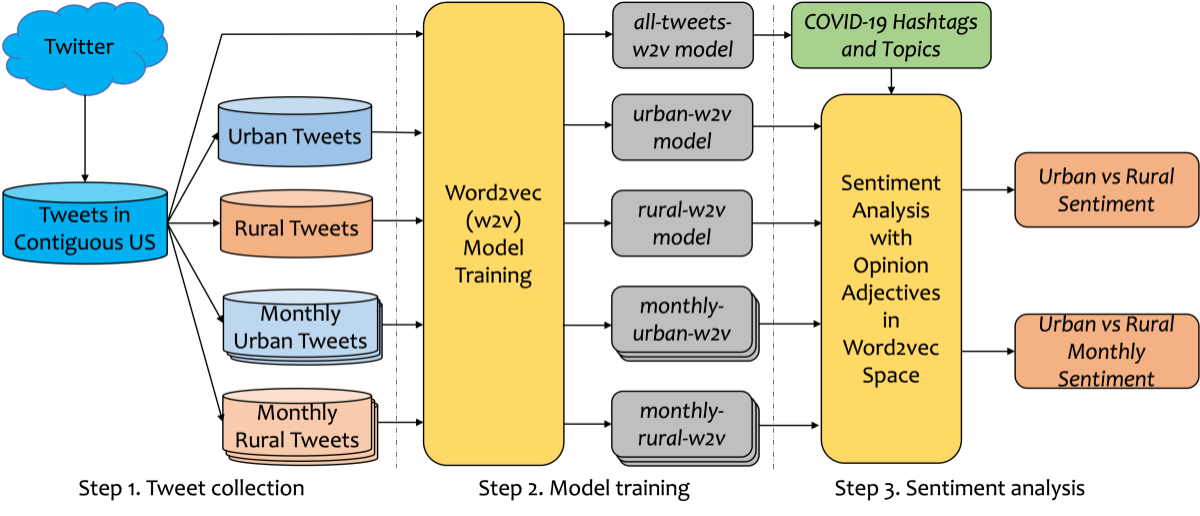
An illustration of the research pipeline. w2v: word2vec.

Figure 1 depicts the data processing and research pipeline for this study. It consisted of three primary steps: (1) tweet collection, (2) model training, and (3) sentiment analysis. The collection step involved the gathering of tweets and a designation of their urban or rural status. The model training step involved training multiple *word2vec* models based on geospatial and timing information. Finally, the sentiment analysis step consisted of COVID-19 topic clustering and multidimensional sentiment analysis with opinion adjectives.

### Data

We used the *Tweepy* python library (version 3.8) to collect 407 million geotagged tweets posted in the contiguous United States through the Twitter application programming interface streaming function between May 2020 and January 2022. A geotagged tweet contains location information as either (1) a specific latitude and longitude or (2) a Twitter place text field. For tweets with the place field, we applied geocoding with the *geopy* python package (version 2.2) to obtain the latitude and longitude, which were then translated into 5-digit zip codes. We did not apply keyword filtering during collection, so the collected tweets are expected to be an unbiased sample of all publicly accessible US geotagged tweets.

#### Urban and Rural Tweet Classification

We mapped each zip code into its respective area type according to its rural-urban commuting area (RUCA) coding [40]. RUCA codes classify US zip codes and census tracts into 10 levels based on commuting information. For example, level 1 stands for a major metropolitan area, while level 10 represents an isolated rural area. These levels can be further grouped into 4 tiers [41,42]: urban core (level 1), suburban (levels 2-3), large rural (levels 4-6), and small town/rural (levels 7-10). In this study, we focused on urban core and small town/rural, as we anticipated more notable differences would be found at these levels.

#### Preprocessing

We removed non-English tweets using the tweet’s *lang* attribute and the *langdetect* language detection package (version 1.0.9). For each remaining tweet, we removed URLs, handlers, and the leading “RT” (which stands for “retweet”). We dropped all punctuation and converted all text into lowercase. We then removed tweets with less than 3 words from the data corpus.

### Methodology

#### Word Embedding

We trained word-embedding models using the skip-gram negative sampling approach implemented in the *gensim* python package (version 4.1.2). We set the vector dimension size to 200 and applied a window size of 5. To characterize sentiment changes among urban and rural users on a monthly basis, we trained *word2vec* models using the monthly corpus with 10 epochs. For efficiency, we trained *word2vec* models using tweets across months for 5 epochs. Parameter tuning was accomplished through word analogy tests (Multimedia Appendix 1 presents the details). We obtained the word embedding model *all-tweets-w2v* from all of the tweets. Two separate models, *urban-w2v* and *rural-w2v*, were generated using all of the urban core and small town/rural tweets, respectively.

#### Topic Extraction With Hashtag Clustering

Twitter users often apply hashtags to label their tweets by topic or theme [43]. Thus, we relied on hashtags to describe and infer topics about COVID-19. We used the word-embedding model *all-tweets-w2v* to find and cluster hashtags related to COVID-19.

The relevance of a hashtag to COVID-19 was measured through a similarity comparison between the given hashtag vector and the vectors for the 3 most common hashtags in the collected data: #covid19, #covid, and #coronavirus. We defined the relevance score as the maximum of the 3 cosine similarity values. We selected all hashtags with a relevance score over a certain relevance threshold and a frequency greater than 50 from the *all-tweets-w2v* model. These hashtags were then subject to an automated clustering process. It should be recognized that the relevance threshold is crucial to our analysis. A larger threshold will lead to a small set of hashtags, resulting in an undersampling of all related hashtags, whereas a smaller threshold will include non–COVID-19 related hashtags. To determine an appropriate relevance threshold, we instructed 5 human annotators to review hashtags with similarity scores above a threshold and the corresponding clustering quality to judge whether hashtags under the current threshold were related to COVID-19. We reviewed hashtag candidates for various thresholds, ultimately settling on a value of 0.5. Further details about the human evaluation are provided in Multimedia Appendix 1.

We applied uniform manifold approximation and projection for dimension reduction (UMAP) [44] on the vector representation of the COVID-19–related hashtags to perform dimensionality reduction and mitigate the impact of a high-dimensional system [45]. Clustering was accomplished via hierarchical density-based spatial clustering of applications with noise (HDBSCAN) [46]. We performed a grid search on UMAP and HDBSCAN to find the clustering model with the highest relative validity score, a fast approximation of the density-based cluster validity [47], to evaluate density-based and arbitrarily shaped clusters. The resulting clusters represented topics related to COVID-19. We defined the topic vector as the weighted average of hashtag vectors in the cluster, where the weight is proportional to the count of the hashtag in the corpus. This definition referenced the general usage of word embedding in document representation [48]. All experiments were performed with the *UMAP* (version 0.5.2), *hdbscan* (version 0.8.28), and *sklearn* (version 1.0.2) python packages.

#### Sentiment Analysis With Opinion Adjectives

Opinion adjectives have been adopted to analyze stereotypes through the geometry of word-embedding vectors [49,50]. For example, the vector for the adjective “lucky” is close to the vector for “clover” [49]. For this work, we relied on the annotated adjectives in SentiWordNet 3.0 [51] to quantify people’s sentiments about COVID-19 topics. In SentiWordNet, each word (“w”) has a positive and negative sentiment score: pos() and neg(), respectively. For example, the word “fine” has pos(fine) and neg(fine) scores of 0.625 and 0.125, respectively. We selected adjectives such that each adjective (“a”) was associated with a pos(a) + neg(a) ≥ 0.5 based on the sentiment score distribution of all adjectives in SentiWordNet (Multimedia Appendix 1 provides details).

We assumed that adjectives that are more often used to describe a hashtag would have a higher similarity score with respect to the hashtag than those that are infrequently used. In this regard, the difference in the use of adjectives between urban and rural users can be measured via the difference in the hashtag-adjective similarity scores between urban and rural word-embedding models. For instance, the adjectives used mainly by urban users to describe a COVID-19 topic can be learned from comparing the topic vector to the adjectives in the *urban-w2v* model. Similarly, the preference of adjectives for rural users can be obtained from the *rural-w2v* model. We retained adjectives that appeared in both *urban-w2v* and *rural-w2v* for sentiment calculation.

We combined the topic-adjective similarity score with the sentiment score for adjectives to learn the sentiment for a topic of interest. Formally, given an adjective collection *A*, the sentiment score of an adjective *a* in *A*, represented as *sent (a)*, is defined as pos(a) − neg(a). The raw sentiment score about a target *t* in the *word2vec* model is defined as follows:


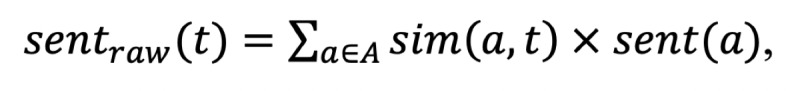


where sim(a,t) refers to the cosine similarity between the vector for adjective *a* and the vector for target *t*.

To enable a comparison between 2 sentiment systems, we normalized the raw sentiment score of topics in each model according to their *z* score, as follows:


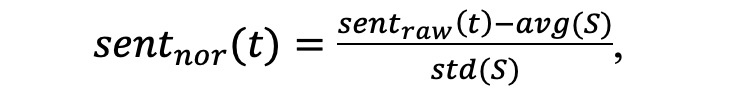


where *S* defines a baseline hashtag set that contains 1000 randomly sampled hashtags. We normalized the urban and rural sentiment scores using 2 different baseline sets in which hashtags were randomly selected from their respective vocabularies. The raw sentiment scores for the baseline hashtags were relied upon to estimate the mean *avg(S)* and standard deviation *std(S).* The resulting normalized sentiment score reflects the magnitude of positive or negative sentiments, which we applied to compare the differences in urban and rural sentiment.

We used a topic vector to represent all of the hashtags in a topic. This approach calculates the sentiment about a topic; however, it cannot estimate the variance across sentiments (ie, the sentiment difference for various hashtags). Thus, for each topic, we sampled 25% of the hashtags without replacement according to their weights (ie, proportional to their counts). We then averaged the vectors for these hashtags to obtain a sampled topic vector. The sentiment score for the sampled topic vector was calculated as described earlier. This process was repeated 10 times to obtain a set of scores, which were used to compute the average sentiments and their variance.

## Results

### Data

**Figure 2:**
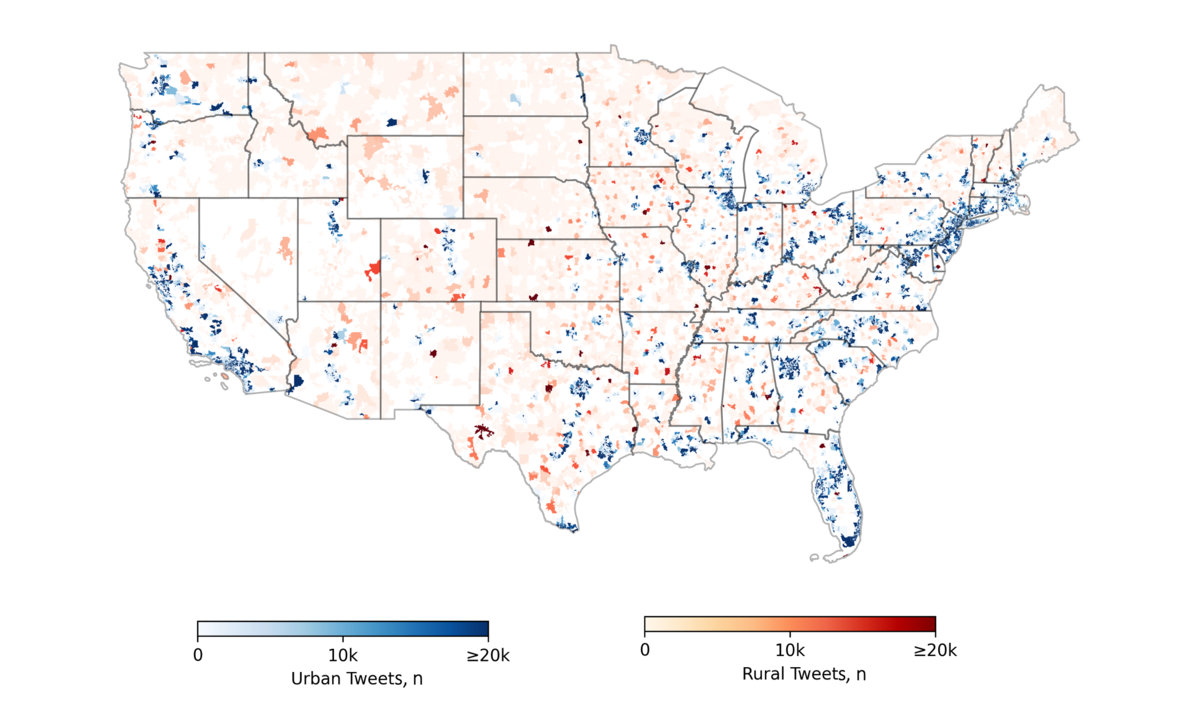
Number of tweets collected in US urban core and small town/rural zip codes.

Figure 2 depicts the number of tweets collected with respect to their region in the United States, where blue represents urban core areas, and red represents small town/rural areas. A darker color means a higher number of tweets in that area. As can be seen in the figure, the distribution generally matches the urban-rural classification scheme in the United States [52]. Table 1 provides summary statistics for the 3 word-embedding models trained using collected tweets.

**Table 1:** Training data for the word-embedding models.

Rural-urban commuting area tier	All tweets	Urban core	Small town/rural
Tweets, n	407 million	350 million	18 million
Words per tweet, n	10.47	10.44	10.54
Unique hashtags, n	474,124	333,177	30,080
Hashtags per tweet, n	0.18	0.18	0.17
*Word2vec* model	*all-tweets-w2v*	*urban-w2v*	*rural-w2v*

### Topic Clustering

**Figure 3:**
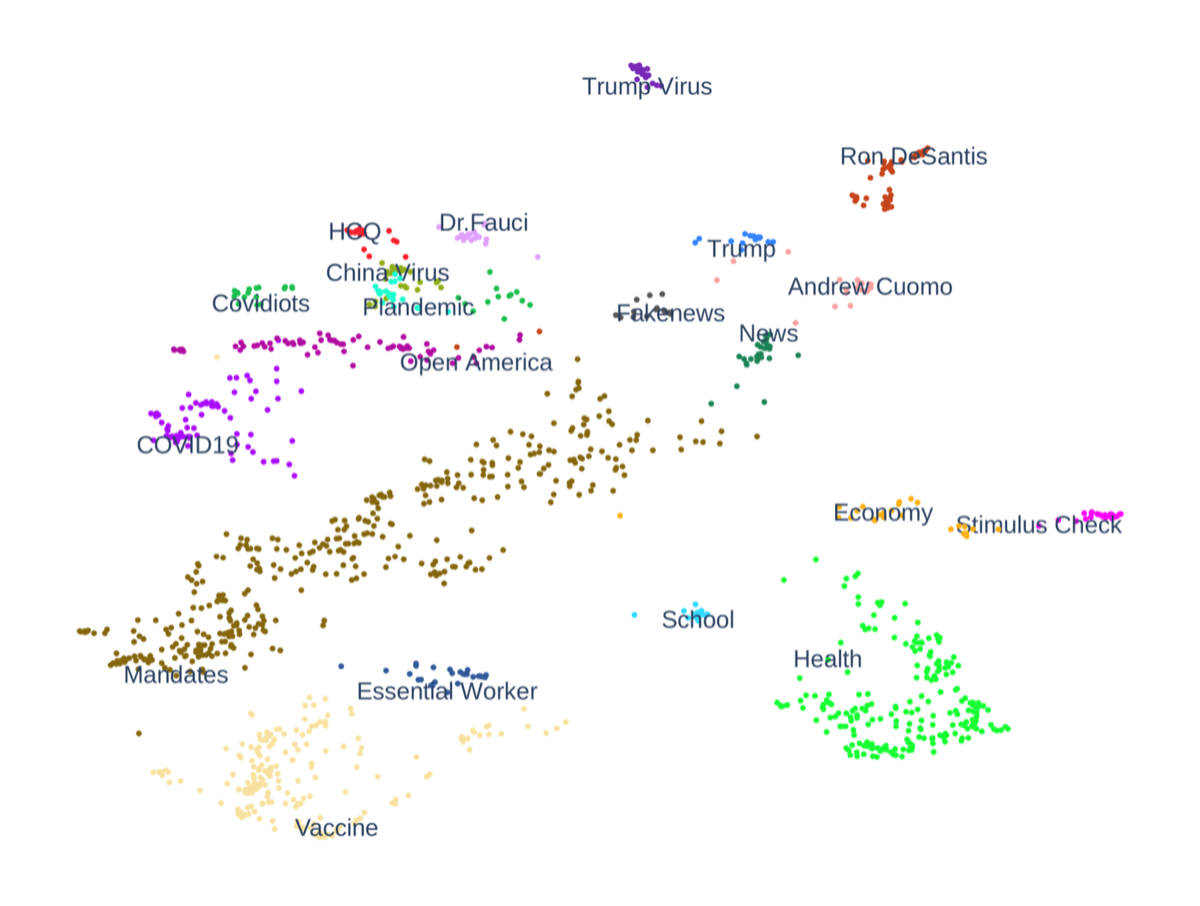
A 2D representation of uniform manifold approximation and projection clustering results for 20 topics. Each point represents a distinct hashtag.

We collected 2666 COVID-19–related hashtags. These hashtags clustered into 30 distinct topics. After a manual review of the clusters, we determined that 20 topics were closely related to COVID-19 in the United States. The other 10 corresponded to less relevant topics, such as general social justice issues (eg, the George Floyd events) and news about the Middle East or COVID-19 in other countries (eg, Canada, India, and Mexico). Figure 3 presents a 2D representation of the word-embedding vectors for the clustered hashtags in the 20 COVID-19–related topics. Based on the closeness of the topic hashtags, we further grouped the topics into 4 categories: misinformation; prevention and treatment; economy; and news and politics. For example, topics belonging to the misinformation category, including “covidiots,” “China virus,” and “plandemic,” appear in the upper left corner. Topics about news and politics are grouped in the upper right corner. Topics in the prevention category and treatment and economy category also exhibit a similar grouping pattern. Specific topics, namely “COVID-19,” “health,” and “school,” do not fall into the 4 categories.

Table 2 shows the number of hashtags, the 10 most tweeted hashtags, and a manually assigned label for each of the 20 topics. It can be seen that the topics “mandates,” “health,” and “vaccine” are affiliated with the most user-generated hashtags, which highlights the users’ concerns about COVID-19 prevention and its impact on health.

**Table 2:** The 20 COVID-19 topics inferred from the tweets collected for this study. The topics are presented in descending order according to the number of unique hashtags they hold. The hashtags are presented in descending order according to their frequency.

Category/topic label		Ten most frequent hashtags	Unique hashtags, n
COVID-19		covid19, coronavirus, covid, covid_19, pandemic, covid-19, corona, covid__19, omicron, covid-19	79
**(1) Misinformation**			
	Open America	openamericanow, nomasks, vaccinemandate, maskmandate, nomask, donotcomply, reopenamerica, vaccinepassport, vaccinepassports, maskmandates	73
	Covidiots	covidiots, antivaxxers, idiots, moron, covididiots, stupidity, morons, antimaskers, antivaxxer, antivax	30
	China Virus	chinavirus, billgates, ccpvirus, wuhanvirus, wuhan, chinaliedpeopledied, chinesevirus, chinaliedandpeopledied, agenda21, wuhancoronavirus	30
	Dr. Fauci	fauci, drfauci, firefauci, faucithefraud, anthonyfauci, fauciliedpeopledied, fauciemails, faucilied, faucifraud, birx	22
	Plandemic	plandemic, hoax, scamdemic, factsnotfear, covidhoax, fearmongering, kungflu, scamdemic2020, fearporn, coronahoax	20
	HCQ	hydroxychloroquine, ivermectin, cnntownhall, remdesivir, hcq, regeneron, hydroxycloroquine, trumpvaccine, hydroxycholoroquine, dexamethasone	16
**(2) Prevention and treatment**			
	Mandates	wearamask, 2020, staysafe, maskup, stayhome, socialdistancing, quarantine, quarantinelife, mask, lockdown	397
	Vaccine	covidvaccine, vaccine, science, getvaccinated, vaccinated, pfizer, moderna, getvaccinatednow, vaccineswork, covid19vaccine	198
	Essential Worker	essentialworkers, nurses, healthcareheroes, inthistogether, healthcareworkers, frontlineworkers, frontlineheroes, healthcareworker, frontliners, frontlines	27
**(3) Economy**			
	Stimulus Check	stimuluscheck, stimulus, unemployment, heroesact, americanrescueplan, stimuluspackage, covidrelief, caresact, covidreliefbill, stimulusbill	28
	Economy	economy, housing, homelessness, unemployed, markets, debt, economic, evictionmoratorium, jobsreport, housingcrisis	26
**(4) News and politics**			
	Ron DeSantis	deathsantis, desantis, rondesantis, gregabbott, deathdesantis, desantisfailedflorida, floridacovidepicenter, harriscounty, floriduh, desantisvariant	58
	Trump Virus	trumpvirus, trumpknew, trumpliesamericansdie, trumpfailedamerica, trumphasnoplan, trumpliedpeopledied, trumpisanidiot, trumpownseverydeath, trumpgate, trumpliespeopledie	31
	News	foxnews, news, cnn, breakingnews, journalism, nytimes, abcnews, nyt, newyorktimes, nbcnews	31
	Andrew Cuomo	cuomo, deblasio, killercuomo, andrewcuomo, governor, chriscuomo, fredo, cuomokilledgrandma, cuomocoverup, governorcuomo	21
	Trump	trump, donaldtrump, potus, whitehouse, realdonaldtrump, presidenttrump, pence, mikepence, potus45, donaldtrumpjr	14
	Fake News	fakenews, lies, factcheck, propaganda, misinformation, conspiracytheory, disinformation, mainstreammedia, factchecking, bantiktok	14
**Other COVID-19–related topics**			
Health		health, cancer, anxiety, depression, publichealth, hiv, diabetes, medicine, doctor, breastcancer	219
School		schools, schoolsreopening, schoolreopening, lausd, stayinformed, reopeningschools, nycdoe, publicschools, virtualuntilsafe, dpa	17

**Figure 4:**
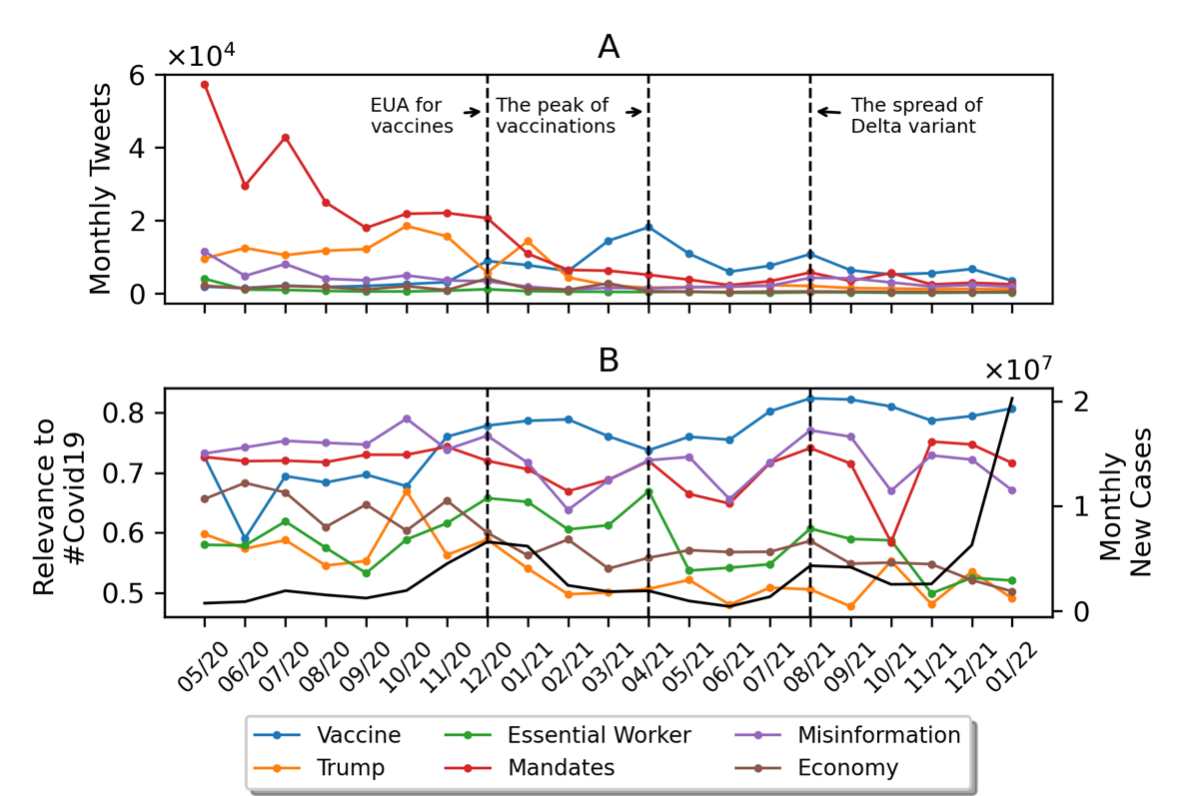
The monthly trend in volume (A) and relevance to COVID-19 (B) for selected topics and categories. The black line indicates the number of monthly new COVID-19 cases in the United States. EUA: emergency use authorization.

Figure 4 shows the trends in volume and relevance for COVID-19 for the selected topics and categories. Specifically, Figure 4A shows the volume of tweets for a topic (number of tweets that contain at least one of the topic hashtags), while Figure 4B shows the relevance of topics to COVID-19. There are several notable observations worth highlighting. First, the most tweeted topic changes over time. For instance, before February 2021, the most tweeted topic was “mandates.” Afterward, “vaccine” became the most tweeted and most relevant topic, with monthly discussions peaking in April 2021. This trend is positively correlated with changes in the number of vaccinated people in the United States. Second, for topics in the “news and politics” category, we found that the changes in the topic “Trump,” both in volume and COVID-19 relevance, are aligned with progress in the 2020 presidential election. The relevance of the “Trump” topic to COVID-19 reached its highest level in October 2020, only a few days before the 2020 presidential election day (November 3), when Donald Trump lost his reelection bid. Third, we observed that before 2022, the trend in the “misinformation” category generally matched the change in the number of new COVID-19 cases. Nevertheless, after 2022, there was a decline in both the volume and relevance scores across all but one topic (“vaccine”), although the number of new COVID-19 cases peaked in January.

### Urban Versus Rural Sentiment

**Figure 5:**
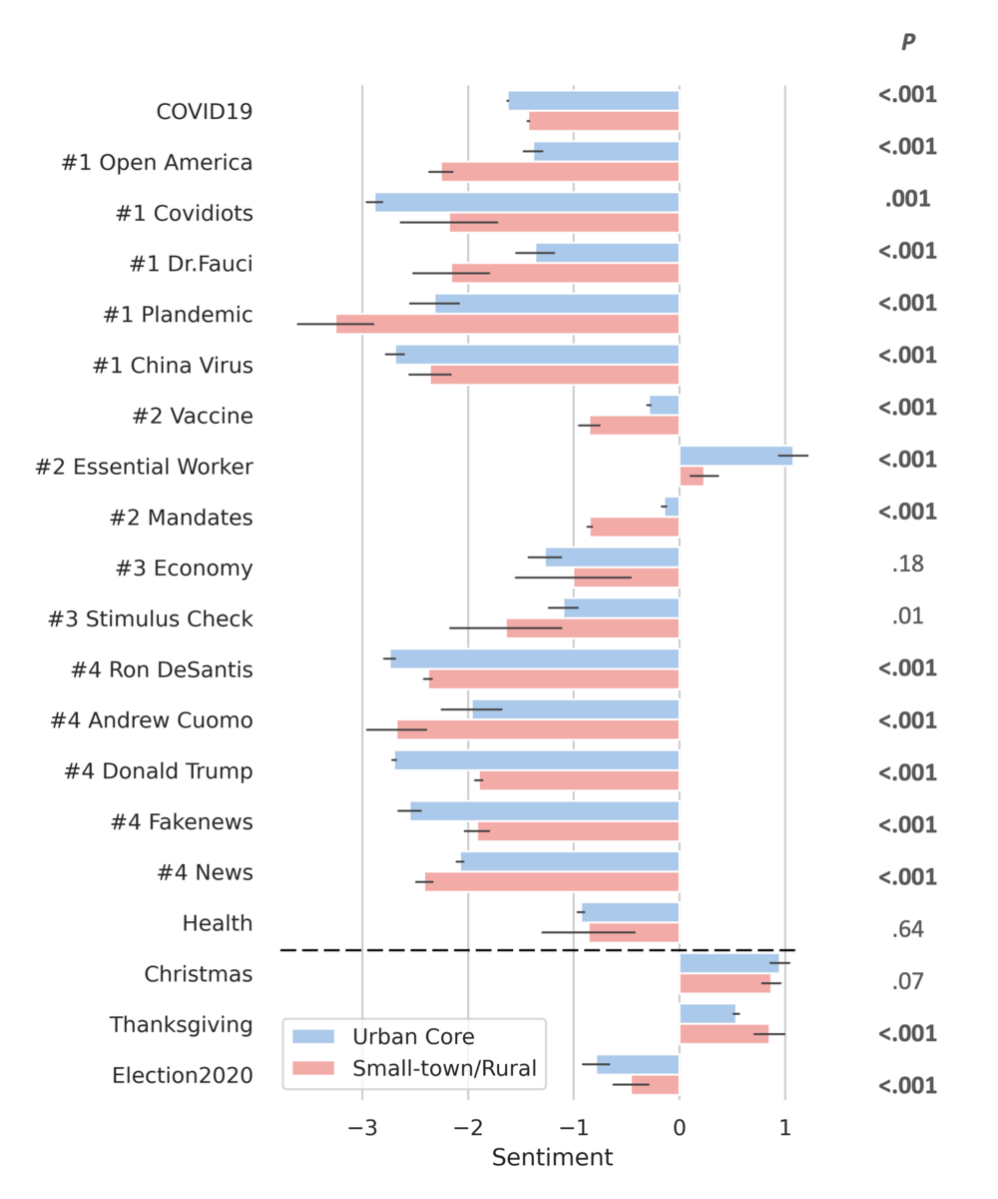
Overall normalized urban and rural sentiment toward COVID-19 and 20 selected topics. The category ID for each topic is shown to the left of the topic name. The error bar indicates SD 1 for the sentiment. The 3 additional topics at the bottom (separated by the dotted lines) are displayed to provide readers with some intuition into the degree of positivity (or negativity) represented by the sentiment score. The raw *P* values from the Welch t tests are shown in the right column; bold text indicates a statistically significant difference (*P*<.05/20) after Bonferroni correction.

Figure 5 depicts the normalized urban and rural sentiments about COVID-19–related topics. We normalized urban and rural raw sentiment scores using the mean (SD) acquired from their baseline hashtag sets. For *urban-w2v*, the mean score was –4.58 (SD 5.84). For *rural-w2v*, the mean score was –11.02 (SD 7.43). Both urban and rural users exhibited negative sentiments for the majority of COVID-19–related topics. The only topic with a positive sentiment was “essential worker.” Both urban and rural users communicated weak negative sentiments (between –1 and 0) for the “mandates,” “vaccine” and “health” topics. By contrast, both groups exhibited a strong negative sentiment (around –2) for the topics “news,” “politics,” and “misinformation.”

For topics related to COVID-19 prevention (i.e., “vaccine” and “mandates”), we observed that rural users had a stronger negative sentiment than urban users. For the topics “misinformation” and “conspiracy theory,” we observed that urban users expressed much stronger negative feelings about the “covidiots” and “fake news” topics, while rural users tended to use adjectives with stronger negative sentiments when discussing “open America,” “plandemic,” and “Dr. Fauci.” Finally, for topics related to politics, we observed a clear political divide when comparing the urban and rural users on their sentiment toward political figures. Urban users wrote about Donald Trump and Ron DeSantis (the governor of Florida since January 2019)—both Republicans—with stronger negative sentiments, while rural users were more likely to criticize Andrew Cuomo (the governor of New York from 2011 to 2021)—a Democrat. These urban versus rural sentiment differences in prevention- and politics-related topics are statistically significant (*P*<.001 with Bonferroni correction). This finding seems to align with the growing political divide between urban and rural America. Urban areas tend to be more liberal, with voters supporting Democrats, whereas rural areas tend to be more conservative, supporting Republicans [53].

To gain further intuition into the degree of positivity (or negativity) represented by the sentiment scores, Figure 5 includes 3 additional topics for comparison: “Christmas,” “Thanksgiving,” and “election 2020.” Among these 3 topics, “Christmas” and “Thanksgiving” had positive sentiments, ranging from 0.5 to 0.8, whereas “election 2020” had a negative sentiment of around –0.5.

### Topic Sentiment Temporal Trends

**Figure 6:**
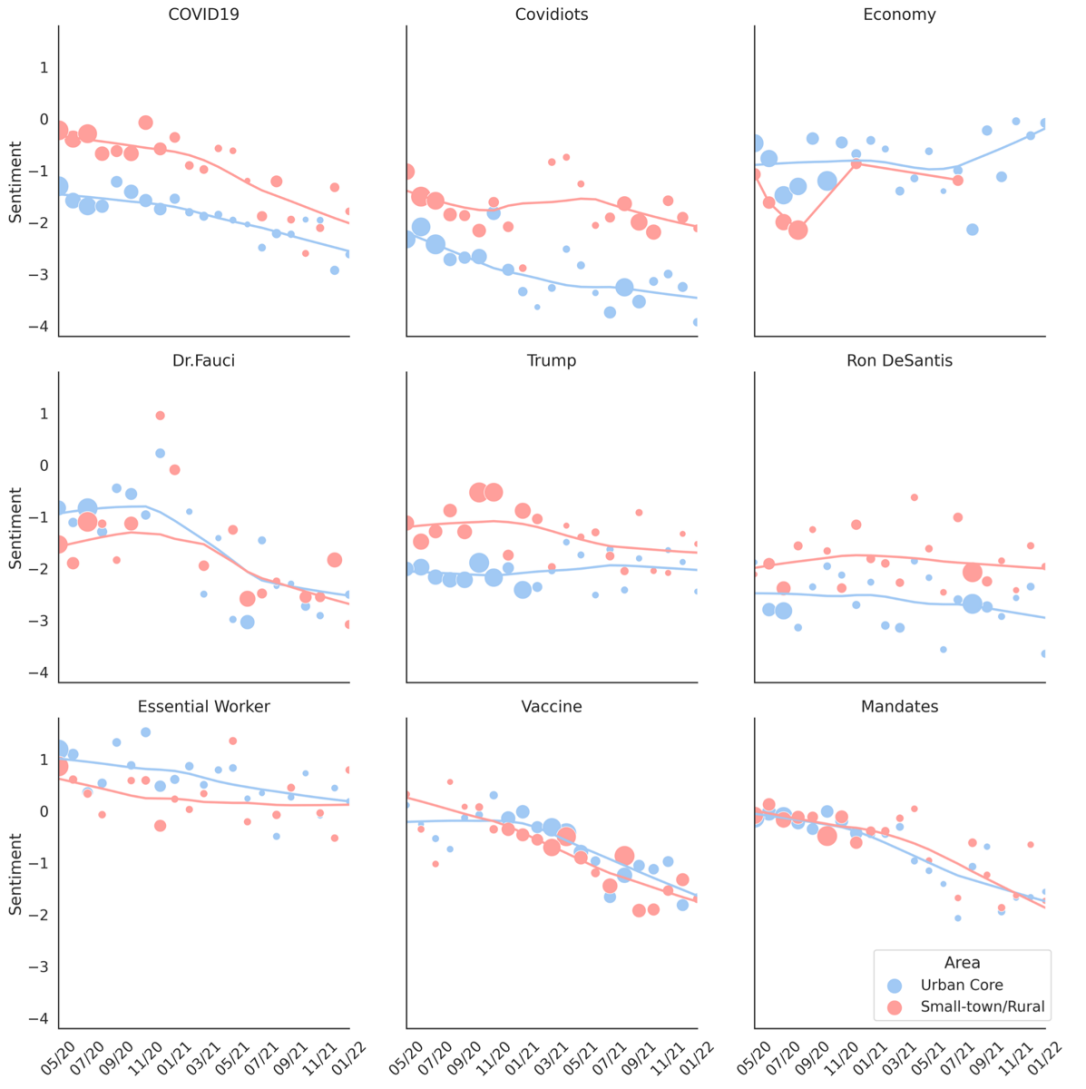
Monthly urban and rural sentiment regarding COVID-19–related topics. For each month, depicted on the x-axis, the center of a dot represents the sentiment value of the topic, while the size of the dot reflects the ratio of the volume of the topic’s current month’s tweets to the sum of the topic’s tweets for all months. The trend lines correspond to a locally weighted linear regression for urban core and small town/rural.

The temporal trends for topic sentiment were characterized as monthly changes in sentiment. However, it should be noted that some topics and their hashtags only appeared in a certain month. For example, in the rural tweets, the hashtags associated with the “school” topic only appeared in July and August of 2020. This may be due to the fact that school start dates in the United States are typically in late August. As a result, we removed the 11 topics with an insufficient number of hashtags or similar urban and rural sentiment trends; we present them in Multimedia Appendix 1. Figure 6 depicts the monthly trend in the sentiments for the 9 remaining topics.

As shown in Figure 6, the attitudes of urban and rural Twitter users regarding COVID-19 gradually became more negative in general. One of the exceptions, shown in the first row in Figure 6, was the “economy” topic, for which urban users appeared to transition from a negative to a positive sentiment. A possible reason for this change is the US economic recovery that started in late 2021 [54]. The second row in Figure 6 shows the sentiment trends for 3 celebrities. For the topic “Dr. Fauci,” December 2020 was a watershed moment in the public’s attitudes about him; this was when he accepted the offer to become the chief medical advisor to the Biden administration. Among politicians, rural users’ sentiments toward Donald Trump and Ron DeSantis were consistently higher than those of urban users. The temporal trends for sentiment about prevention-related topics are depicted in the third row in Figure 6, where urban and rural users show similar, gradually declining trends toward “vaccine” and “mandates.” While rural users had relatively stable sentiments toward the topic of “essential workers,” urban users’ sentiments slowly became negative.

## Discussion

### Principal Findings

There are several notable findings of this investigation. First, we observed that urban and rural users evidently harbor different sentiments about certain COVID-19–related topics. In particular, urban users exhibited stronger negative sentiments about “covidiots,” “China virus,” “economy,” and “fake news.” By contrast, rural users showed stronger negative sentiments toward “plandemic,” “Dr. Fauci,” and prevention strategies (“vaccine” and “mandates”). These findings are consistent with those of prior investigations [4,6]. Callaghan and colleagues [6] found that rural residents were less likely to “participate in several COVID-19-related preventive health behaviors,” and Chauhan and colleagues [4] observed that rural residents were less concerned about the coronavirus. Moreover, we observed a clear political divide between urban and rural users through the sentiment analysis of 3 politicians. For instance, during the time window covered in this study, urban users viewed Andrew Cuomo more favorably than Donald Trump or Ron DeSantis, while the opposite could be said for rural users. These findings are also consistent with studies on political polarization [55]. All of these findings provide evidence that, with our proposed model, social media data can be effectively leveraged to gain timely insight into the public understanding of and sentiment toward hot social events.

At the same time, we believe that the approach for studying public sentiment introduced in this work has several benefits over prior methods. First, by combining the word-embedding models with sentiment-rich opinion adjective lexicons, users of this approach can conduct sentiment analysis in the learned semantic vector space. This allows users to directly infer the sentiments of a population group toward a topic. In comparison to tweet-level sentiment analysis, one advantage of this approach is that it does not require identifying COVID-19–related tweets by using either keyword filters or machine learning classifiers; thus, this approach is more robust against noise (eg, misspellings, synonyms, and abbreviations) in the online data. Second, unlike commonly used topic modeling techniques such as latent Dirichlet allocation, this new method uses word-embedding vector clustering to identify hashtags and topics of public interest, which works well on large amounts of noisy short-text data, such as in tweets. Third, while our approach was tailored for a sentiment analysis of COVID-19, we believe that the trained word-embedding models can be directly used for sentiment analysis of other social events without the hassle of a new round of data collection and labeling. For instance, our data collection period covers the time of the 2020 presidential election; thus, the trained model can be directly used for election-related sentiment analysis. Another possible application of this model would be to build a topic extraction and sentiment analysis platform where users can input any event of interest to obtain related topics and to infer the public’s sentiments about the event in rural or urban areas. Our learned word-embedding models are publicly available on GitHub [56].

### Limitations

There are several limitations to this study, which we believe serve as opportunities for future research. First, we relied on the tweets’ place attribute to obtain the users’ geolocation and to infer the users’ urban or rural status. This step is not completely accurate, as there are several “nonformatted” places in the collected tweets. The nonformatted places can be ambiguous, such as “McDonald’s,” or too general, such as “Iowa, USA.” Through a manual review of 200 randomly sampled tweets, we found 19 (10%) tweets with nonformatted place attributes. The geocoding results of tweets with a nonformatted place attribute may make our results less significant than the actual urban versus rural differences. Second, to quantify the sentiment of a particular group, our method requires training a word-embedding model for that group. Our method is less effective if the goal is to compare multiple social groups with different demographics. This issue may be resolved with word-embedding geometry [57]: performing sentiment analysis of the subspace of the aspect of interest. Finally, it should be recognized that social media–based investigations can, at times, be limited by population sampling bias [58], such that the results may not generalize to the entire US population. For example, it has been shown that Twitter users are more likely to be younger and lean politically to the left than the general public [59]. However, we believe that when faced with emerging social issues, social media–based sentiment analysis can broadly indicate the public’s views, opinions, and needs. In other words, social media analysis can serve as a timely and complementary approach to inform policy making and resource allocation.

### Conclusions

This study introduces a novel approach to characterize the public’s sentiment about COVID-19 and related topics. By applying topic recognition and subsequent sentiment analysis, we discovered a clear difference between urban and rural users in their sentiments about COVID-19 prevention strategies, misinformation, politicians, and the economy. While these findings might not be representative of the sentiment of the American public more broadly, we believe that such investigations could help policy makers obtain a more comprehensive understanding of the sentiment differences between urban and rural areas on COVID-19 and related topics, so that more targeted deployment of epidemic prevention efforts can be made. Finally, we wish to highlight that our approach is not limited to COVID-19, and it can readily be extended to other topics of interest without additional data collection or model training.

## Appendix

Multimedia Appendix 1 Word Embedding Model Parameter Selection, Selection of Relevance Threshold of COVID-19 Related Hashtags, and Opinion Adjectives Selection.

## Abbreviations

HDBSCAN: hierarchical density-based spatial clustering of applications with noise
RUCA: rural-urban commuting area
UMAP: uniform manifold approximation and projection
