# Supplementary material for: Examining Rural and Urban Sentiment Difference in COVID-19–Related Topics on Twitter: Word Embedding–Based Retrospective Study
Source: J Med Internet Res. 2023 Feb 15;25:e42985. doi: 10.2196/42985 (PMC9937112; doi:10.2196/42985)
Supplement: Multimedia Appendix 1 [file jmir_v25i1e42985_app1.docx]

Supplemental Material for “Examining Rural and Urban Sentiment Difference in COVID-19–Related Topics on Twitter: Word Embedding–Based Retrospective Study”

Yongtai Liu, PhD^1^, Zhijun Yin, PhD^1,2^, Congning Ni, ME^1^, Chao Yan, PhD^2^, Zhiyu Wan, PhD^2^, Bradley A. Malin, PhD^1,2,3^

^1^Department of Computer Science, Vanderbilt University, Nashville, TN;

^2^Department of Biomedical Informatics, Vanderbilt University Medical Center, Nashville, TN;

^3^Department of Biostatistics, Vanderbilt University Medical Center, Nashville, TN

Corresponding Author:

Yongtai Liu, PhD

2525 West End Ave. Suite 8058, Nashville, TN, 37203

Department of Computer Science, Vanderbilt University

Email: yongtai.liu@vanderbilt.edu

## Section A. Word Embedding Model Parameter Selection

The hyperparameters of the word2vec models that were tuned in this study are the vector size, window size, and the number of iterations. These hyperparameters were selected based on a previous study of word2vec [1]. We used one month of collected tweets (2021-05) as the text corpus to train different word embedding models by using grid search. Then, a word analogy test was performed to select the appropriate parameters for further word embedding model training.

For the word analogy test, we followed the work of Mikolov et al.[2] The results are shown in Figure 1.


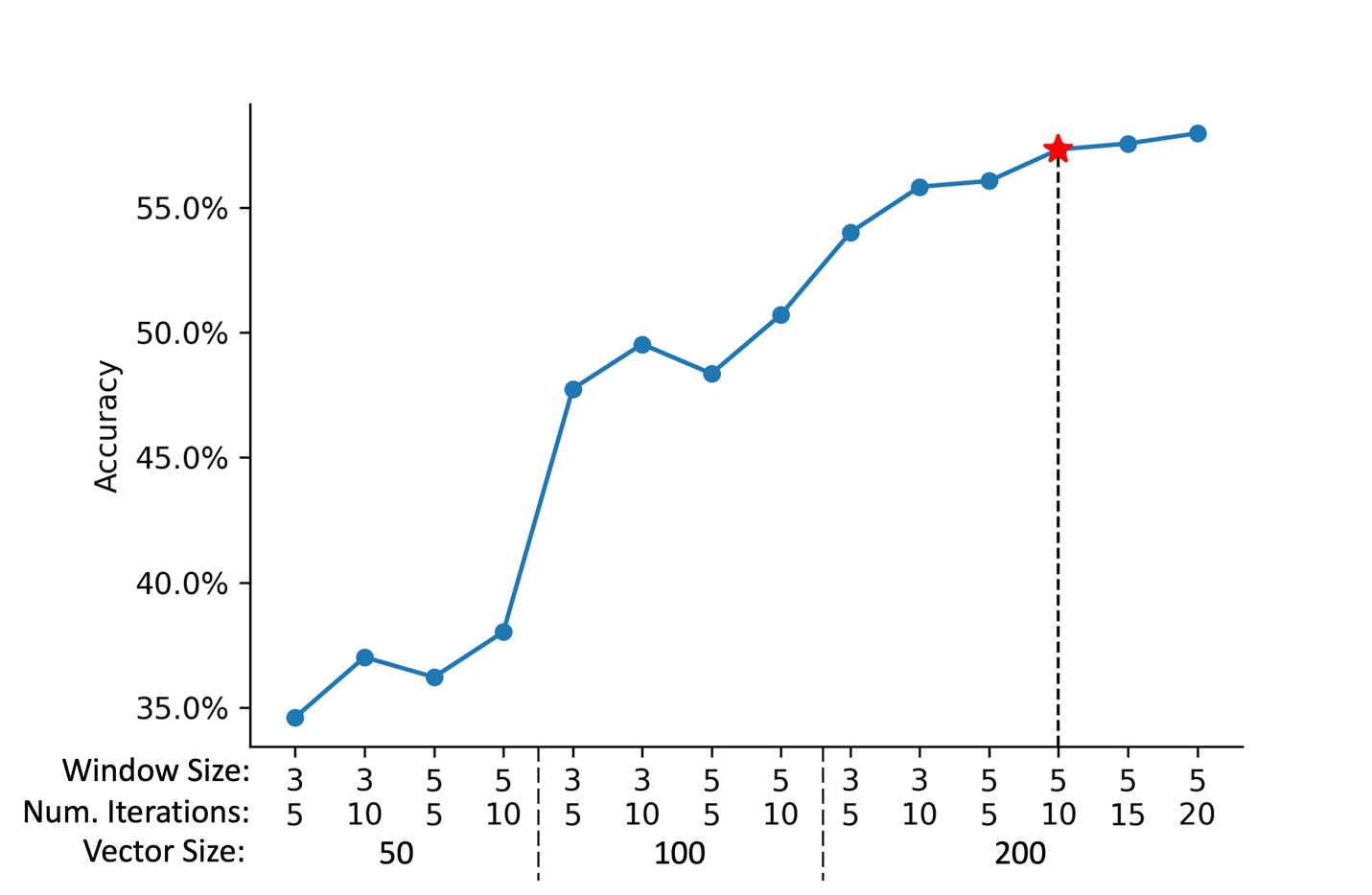


Figure 1. Word analogy test accuracy for 14 different parameter settings. The first row in x-axis shows the window size, the second row shows the number of trained iterations, the third raw shows the selected vector size. The y-axis represents the analogy test accuracy.

As shown in Figure 1, increasing vector size had the most remarkable improvement in test accuracy among the three candidate hyperparameter values. Thus, the vector dimension was set to 200, a common choice in Twitter word embedding training [3]. As for window size, we limited the maximum window size to 5. This is because the average number of words per tweet in our collected data was 10.44; setting a window size to greater than 5 implies that the vector of the current word depends on a word from the words in another tweet, which is unreasonable. Based on experiments result, we set the window size to 5. Finally, a comparison of experiments with vector size of 200 (the rightmost six dots in Figure 1) showed that training the corpus more than ten times had limited improvements, the gain of the accuracy was quite small after the hyperparameters of (5, 10, 200). Considering the model training time, we set the number of iterations to 10 for the monthly corpus.

## Section B. Selection of Relevance Threshold of COVID-19 Related Hashtags

We relied on human evaluation to determine the relevance threshold from a candidate list of [0.4, 0.45, 0.5, 0.55]. To determine an appropriate relevance threshold, we directed five human annotators to review hashtags above a threshold and the corresponding clustering quality to judge whether hashtags under the current threshold are related to COVID-19. We reviewed hashtag candidates with respect to thresholds 0.4, 0.45, 0.5, and 0.55, ultimately set on a value of 0.5.

The first round of human evaluation focused on the “recall” or the quantity of collected hashtags, and the aim was to find more COVID-19 related hashtags, this round was accomplished by one annotator. For each threshold *t*, we first defined a score range, [*t*, *t*+0.05). Then we asked the annotator to check the 100 randomly selected hashtags with a relevance score in the given score range and label whether the hashtags are related to COVID-19. The results are shown in Table 2.

Table 2. Number of COVID-19 related hashtags in random samples at different threshold level, labeled by one annotator.

| ID | Threshold | Score range | Number of COVID-19 related hashtags in 100 randomly sampled hashtags | Example hashtags |
| --- | --- | --- | --- | --- |
| 1 | 0.55 | [0.55, 0.59) | > 70 | faucihero, unmaskamerica |
| 2 | 0.5 | [0.50, 0.55) | 40~50 | firefauci, quarentinelife |
| 3 | 0.45 | [0.45, 0.50) | 10~20 | maskupnola, backtoschool2020 |
| 4 | 0.4 | [0.40, 0.45) | < 5 | coronavirussicilia, backtobusiness |

From Table 2, it can be seen that there are still some COVID-19 relevant hashtags below threshold 0.55, setting a threshold equal to or higher than 0.55 will result in an incomplete collection of COVID-19 relevant hashtags. Therefore, in the second round of human evaluation, we removed 0.55 from the threshold candidates.

The second round of human evaluation focused on the “precision”, or the quality of collected hashtags. Specifically, for each threshold, we collected all relevance hashtags and clustered them into various clusters. Then, we asked five annotators to review the generated clusters, and to judge 1) whether a given cluster is related to COVID-19, and 2) the quality of the given cluster in terms of the similarity of hashtags within the cluster. Each cluster was judged by three annotators independently. The questionnaire is shown in the textbox below.

*Each table contains all clusters generated from the given hashtags and the top ten hashtags (sorted by counts) for each cluster.*

*For each cluster, please answer the following two questions with provided****dropdown list****:*

*Q1. Based on the hashtags, is this cluster related to covid-19?*

*Possible Answers: Yes/Maybe/No*

*If the answer to****question 1 is Yes or Maybe****, please continue to answer question 2:*

*Q2. What is the quality of the cluster? (i.e., are all ten hashtags related to a certain topic? Or are the ten hashtags related to different topics?)*

*Possible Answers: Good / OK / Bad (corresponding scores: 2/1/0)*

For clusters related to COVID-19 (clusters with an answer yes or maybe for question 1), we report reviewers’ Kappa agreement on question 1 and average cluster quality score in Table 3.

Table 3. Human evaluation of hashtag cluster quality.

| Id | Threshold | Fleiss' kappa of Q1 on COVID-19 related clusters | Average clusters’ quality score (Q2) |
| --- | --- | --- | --- |
| **1** | **0.50** | **0.364** | **1.545** |
| 2 | 0.45 | 0.253 | 1.487 |
| 3 | 0.40 | 0.340 | 1.483 |

Since threshold 0.5 resulted in the highest kappa agreement score and the highest quality score, we selected 0.5 as the relevance threshold for the further analysis.

## Section C. Opinion Adjectives Selection

In SentiWordNet 3.0, each adjective, say *a*, has multiple meanings, and each meaning has a *pos(a)* and *neg(a)* score. For example, the word “unable” has three meanings, unable (#1), unable (#2), and unable (#3). The numbers are ordered based on the frequency of use. The most common meaning (#1) is “not having the necessary means or skill or know-how”, and the other two meanings (#2, #3) are “lacking necessary physical or mental ability” and “lacking in power or forcefulness”. These three meanings have different sentiment scores. For instance, unable (#1) has a positive score of 0.0 and a negative score of 0.75, whereas unable (#2) has scores of 0.0 and 0.375, respectively. In this paper, we only focused on the *pos(a)* and *neg(a)* scores for *a*(#1). The *pos(a) + neg(a)* score distribution for all adjectives in SentiWordNet3.0 are shown in Figure 1.


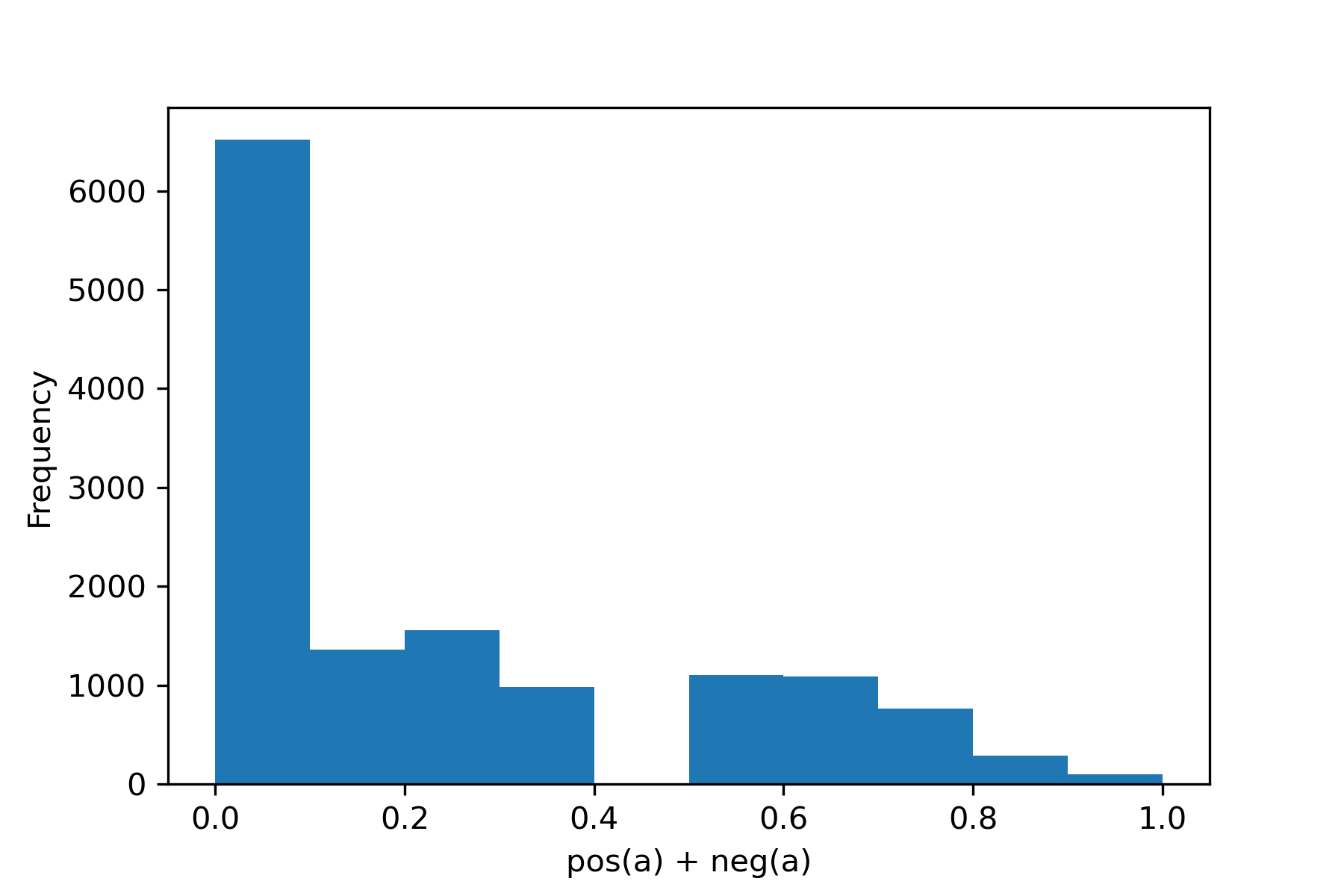


Figure 1. The pos(a) + neg(a) score distribution for all adjectives (a) in SentiWordNet3.0

As shown in Figure 1, there are two groups of adjectives: the adjectives with *pos(a) + neg(a)* ≥ 0.5 and the adjectives with *pos(a) + neg(a)* < 0.5. We considered the adjectives with *pos(a) + neg(a)* ≥ 0.5 as “sentiment-rich” adjectives and kept them in the further sentiment analysis.

## Section D. Monthly urban and rural sentiment regarding 20 COVID-19 related topics


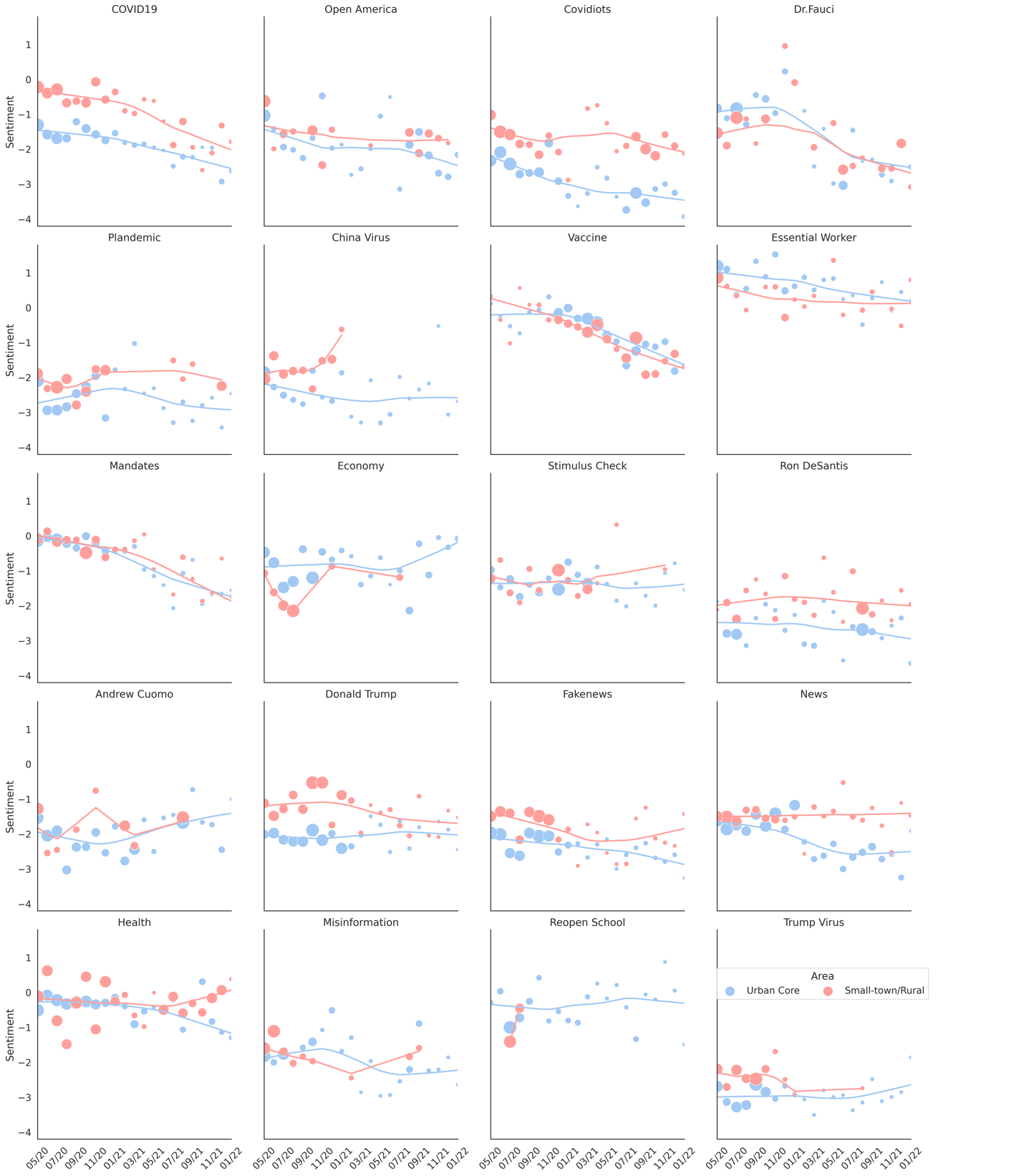


Figure 2. Monthly urban and rural sentiment regarding 20 COVID-19 related topics.

## Reference

1. Yang X, Macdonald C, Ounis I. Using word embeddings in Twitter election classification. *Inf Retr J*. 2018;21(2):183-207. doi:10.1007/s10791-017-9319-5

2. Mikolov T, Sutskever I, Chen K, Corrado GS, Dean J. Distributed Representations of Words and Phrases and their Compositionality. In: *Advances in Neural Information Processing Systems*. Vol 26. Curran Associates, Inc.; 2013. Accessed September 8, 2022. https://proceedings.neurips.cc/paper/2013/hash/9aa42b31882ec039965f3c4923ce901b-Abstract.html

3. stanfordnlp/GloVe. Published online September 8, 2022. Accessed September 8, 2022. https://github.com/stanfordnlp/GloVe
